# The Structures, Spectroscopic Properties, and Photodynamic Reactions of Three [RuCl(QN)NO]^−^ Complexes (HQN = 8-Hydroxyquinoline and Its Derivatives) as Potential NO-Donating Drugs

**DOI:** 10.1155/2018/7029376

**Published:** 2018-12-02

**Authors:** Leilei Xie, Lifang Liu, Wenming Wang, Zhiou Ma, Liqun Xu, Xuan Zhao, Hongfei Wang

**Affiliations:** ^1^Key Laboratory of Chemical Biology and Molecular Engineering of Education Ministry, Institute of Molecular Science, Shanxi University, Taiyuan 030006, China; ^2^Key Laboratory of Energy Conversion and Storage Materials of Shanxi Province, Institute of Molecular Science, Shanxi University, Taiyuan 030006, China; ^3^Department of Chemistry, University of Memphis, Memphis, TN 38152, USA

## Abstract

The structures and spectral properties of three ruthenium complexes with 8-hydroxyquinoline (Hhqn) and their derivatives 2-methyl-8-quinolinoline (H2mqn) and 2-chloro-8-quiolinoline (H2cqn) as ligands (QN = hqn, 2mqn, or 2cqn) were calculated with density functional theory (DFT) at the B3LYP level. The UV-Vis and IR spectra of the three [RuCl(QN)NO]^−^ complexes were theoretically assigned via DFT calculations. The calculated spectra reasonably correspond to the experimentally measured spectra. Photoinduced NO release was confirmed through spin trapping of the electron paramagnetic resonance spectroscopy (EPR), and the dynamic process of the NO dissociation upon photoirradiation was monitored using time-resolved infrared (IR) spectroscopy. Moreover, the energy levels and related components of frontier orbitals were further analyzed to understand the electronic effects of the substituent groups at the 2nd position of the ligands on their photochemical reactivity. This study provides the basis for the design of NO donors with potential applications in photodynamic therapy.

## 1. Introduction

The structure and reactivity of transition-metal-NO complexes have gained significant interest in recent years because of the important role of nitric oxide (NO) as a signaling molecule in biological systems [1–5]. NO plays important functions in various physiological processes [6–9]. Moreover, the active centers of several important biological enzymes contain metal ions bound with the NO ligand; therefore, studies of the structures and spectra of metal-NO complexes are important to understand the dynamic reactivity and their functions.

The utility of ruthenium (Ru) complexes to design potential anticancer drugs and cellular imaging agents has been extensively investigated [10–15]. Compared to iron-based nitrosyl complexes, Ru nitrosyls are promising candidates as potential NO-donating agents for targeted delivery of NO to physiological targets due to their inherent stability and modest photosensitivity [16–21]. Detailed structural and spectroscopic analyses of Ru complexes with different ligands are essential to investigate the kinetic process of the photoreaction. These studies provide a foundation to control NO release at the physiological target.

**Figure 1:**
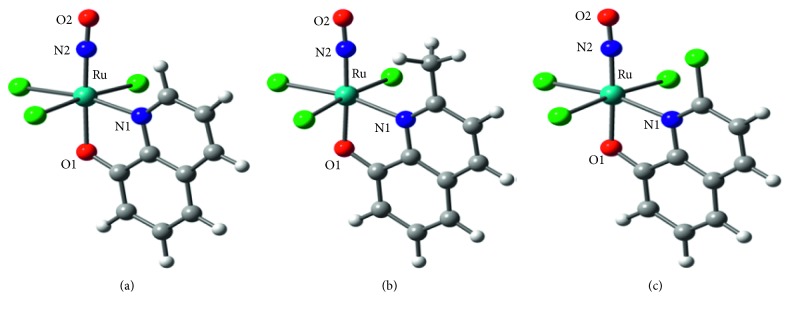
Optimized structures of [RuCl_3_(hqn)NO]^−^ (a), [RuCl_3_(2mqn)NO]^−^ (b), and [RuCl_3_(2cqn)NO]^−^ (c) complexes.

A series of nitrosylruthenium (Ru-NO) complexes with polypyridyl complexes have been reported. They are coordinated with (N,N) bidentate ligands forming cationing complexes. The cytotoxicity against tumor cell and their vasodilation effects have been studied [20–25]. Here, three [RuCl_3_(QN)(NO)]^−^ anionic complexes were synthesized using 8-hydroxyquinoline and its derivatives (HQN) as ligands. These ligands are bidentate chelators that bind metal ions via O-N atoms.  Figure 1 shows the structures of the [RuCl_3_(QN)(NO)]^−^ complexes and HQN ligands (HQN = 8-hydroxyquinoline (Hhqn), 2-methyl-8-quinolinol (H2mqn), and 2-chloro-8-quinolinol (H2cqn)). DFT calculations allowed the assignment of bands observed in the electronic and IR spectra of the complexes. Furthermore, the behavior of the three complexes upon photoirradiation was investigated using time-resolved spectroscopy technology. The electronic structures and molecular orbitals of these complexes were calculated to better understand the electronic effect of the substituted group at the 2nd position of the ligands. This study provides insight into the photodynamic properties and potential applications of the nitrosylruthenium (II) complexes.

## 2. Experimental

### 2.1. Synthesis

Chemical reagents and solvents were purchased from Sigma (St. Louis, MO, USA) and local vendors. The complexes were synthesized according to a previously described method with modifications [26, 27] and characterized by ^1^H NMR spectroscopy using a Bruker 600 MHz spectrometer.

### 2.2. Spectra Measurements

After the complexes were dissolved in DMSO, the UV-visible spectra were recorded on a Thermo 220 spectrophotometer. The IR spectra were measured on an IS50R FT-IR spectrometer (Thermo Fisher) from 2000 to 1400 cm^−1^ at 1 cm^−1^ resolution. The sample solutions were added to an IR cell composed of two CaF_2_ windows (25 mm in diameter and 2 mm thick), which were separated by an O-shaped 50 *μ*m thick Teflon spacer.

The photoreaction kinetics was monitored via the IR spectra as a function of irradiation time. The IR spectra were recorded simultaneously for 30 min in the CaF_2_ windows while being irradiated with a fiber connected to an Xe lamp with 420 nm band-pass filter (0.2 W/cm^2^).

The electron paramagnetic resonance (EPR) spectra were obtained using a Bruker ESP-500E spectrometer at 9.8 GHz, X band, with 100 Hz field modulation. The three complexes (5 mM) mixed with 5 mM Fe(MGD)_2_ were quantitatively injected into quartz capillaries, respectively. The sample was then illuminated in the cavity of the EPR spectrometer with an Hg lamp (LOT-QuantumDesign GmbH) at 365 nm. All experiments were performed at room temperature (20°C).

### 2.3. Quantum Chemical Calculations

Gaussian 09 and Gaussview 5 program packages were used for calculations and structure visualization, respectively [28, 29]. The original models for the three complexes were built based on the crystal structure of [(CH_3_)_4_N][RuCl(2cqn)NO] complex [27]. All structures were fully optimized with Becke's three-parameter hybrid functional and the Lee–Yang–Parr correlation functional (B3LYP) [30–32] in the DMSO solvent. The basis sets aug-cc-pVDZ-PP and 6-311++G(d,p) were used to describe the Ru atom and the ligand atoms, respectively [33, 34]. The charge was set to −1, and both *S* = 0 and *S* = 1 states for the complexes were optimized.

The UV-visible spectra for the three complexes in DMSO solution were simulated with a time-dependent (TD-DFT) method, respectively, and the solvent effect was considered via the polarization continuum model [35, 36]. The natural atomic charges and Wiberg bond index of the complexes were obtained by natural population analysis (NPA) and natural bond orbital (NBO) analysis [37, 38].

## 3. Results and Discussion

### 3.1. Molecular Geometry

The selected and calculated bond lengths and the angles for three complexes are listed in Table 1. Most of the calculated bond lengths and angles of the optimized geometries (Table 1) deviate from crystal structural data by 0.03 Å and 2°, respectively. The theoretical bond lengths of Ru-N2, Ru-O1, and N2-O2 in [RuCl_3_(2cqn)NO]^−^ complex deviate the experimental data less than 0.01 Å, which is near to the uncertainty caused by the experiment measurements [39, 40].

The structures for both *S* = 0 and *S* = 1 as potential ground states were optimized, respectively. As shown in Table 2, the angles of the Ru-NO in the optimized structures of the lowest triplet excited states for three complexes are 143.2, 177.1, and 177.4 degrees, respectively. It is worth noting that the Ru-NO is in the bending model for [RuCl_3_(hqn)NO]^‒^ in the triplet excited states. However, they are linear for both [RuCl_3_(2mqn)NO]^−^ and [RuCl_3_(2cqn)NO]^−^ in the singlet state and the lowest-triplet excited states. The calculated energy of the singlet state is the lowest one, suggesting the complexes with diamagnetic ground states.

### 3.2. Molecular Orbital Analyses

**Figure 2:**
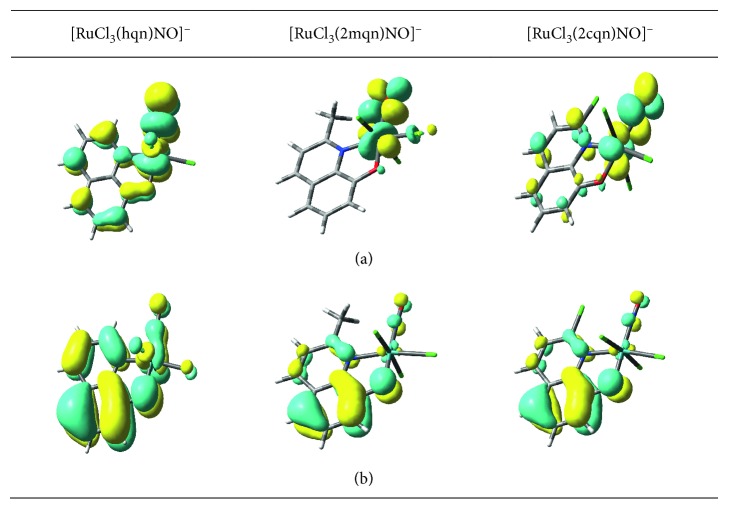
Contour diagrams of the calculated LUMO (a) and HOMO (b) of three complexes. Negative values of the wave function are represented in yellow.

The HOMO-LUMO interactions were calculated to probe the reactivity of the various molecular systems [41–45]. The contour plots of the frontier orbitals for three complexes are shown in Figure 2, and the calculated HOMO and LUMO energy levels are shown in Table 2. The calculations were performed with the DMSO solvent. In the three complexes, the HOMO is described as a QN ligand-based orbital that contains some Ru (*d*) and NO (*p*) character, while the LUMO contains an antibonding overlap of the Ru (*d*) and *π*^*∗*^ NO (*p*) orbitals. It suggests that the (Ru(II)-NO^+^) group plays an important role in the photochemical reaction of nitrosylruthenium (II) complexes containing 8-quinoliolate and its derivatives.

For the [RuCl_3_(2mqn)NO]^−^ complex, the HOMO and LUMO relative orbital energies are higher and its LUMO-HOMO gap is smaller than those of [RuCl_3_(hqn)NO]^−^ complex. However, the HOMO and LUMO relative orbital energy is lower for [RuCl_3_(2cqn)NO]^−^ complex, while its LUMO-HOMO gap is larger than [RuCl_3_(hqn)NO]^−^. The variation of HOMO and LUMO energy orbitals suggests that different substituted groups in the 2nd ligand position could adjust the relative energies of the front orbitals and could affect the stabilities and reactivity of these complexes.

### 3.3. Electronic Absorption Spectra

**Figure 3:**
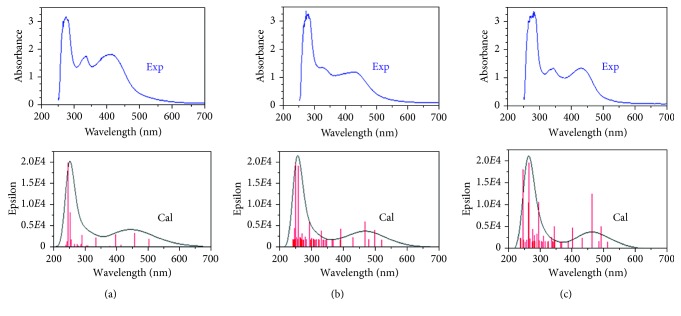
Recorded and calculated electronic absorption spectra of [RuCl_3_(hqn)NO]^−^ (a), [RuCl_3_(2mqn)NO]^−^ (b), and [RuCl_3_(2cqn)NO]^−^ (c) complexes (blue: experimental; black: calculated; red: calculated oscillator strength).

The UV-visible absorption spectra of the three [RuCl_3_(QN)NO]^−^ complexes in DMSO are shown in Figure 3. These three complexes have similar absorption curves in the ultraviolet and visible region with a 11 nm shift in the absorption peak and a 21 nm shift in the absorption peak between [RuCl_3_(2mqn)NO]^−^ and [RuCl_3_(2cqn)NO]^−^ in the ultraviolet region, respectively. In the visible region, the maximum absorption band is at 415 nm for [RuCl_3_(hqn)NO]^−^, 424 nm for [RuCl_3_(2mqn)NO]^−^, and 430 nm for [RuCl_3_(2cqn)NO]^−^.

In the UV region, the three complexes display absorption bands at 274 and 337 nm, 270 and 323 nm, 281 and 344 nm, respectively. The corresponding calculated values are 250 and 336 nm, 255 and 331 nm, 262 and 346 nm, respectively. The calculated wavelengths have an error of less than 24 nm compared to the experimental data from the TDDFT method while taking into account of the solvent effect.

The lowest-energy peak near 430 nm predominates the HOMOs-LUMOs excitation in the visible region. The absorption peaks for three complexes were calculated to be near 441, 467, and 461 nm, with deviation from the experimental value by about 30 nm. Analysis of the electronic structures and orbital components of the complexes indicates that these absorption bands mainly originate from the d(Ru)*π* + *p*(QN and Cl ligands) ⟶ (d(Ru) + *p*^*∗*^(NO and QN ligands)) charge transfer processes, which were labeled as MLCT and LMCT processes (L stands for NO, Cl, and QN ligands).

### 3.4. Infrared Spectra

**Figure 4:**
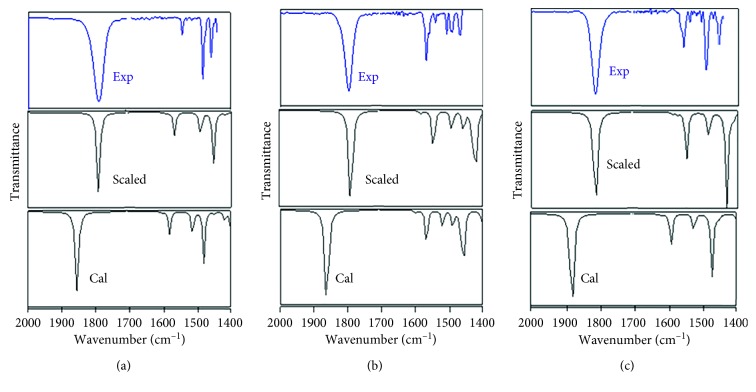
Recorded and calculated IR spectra of [RuCl_3_(hqn)NO]^−^ (a), [RuCl_3_(2mqn)NO]^−^ (b), and [RuCl_3_(2cqn)NO]^−^ (c) complexes in the 2000–1400 cm^−1^ region (blue: experimental; black: calculated).

Figure 4 shows the infrared spectra of the three complexes recorded in DMSO. For comparison, the experimentally observed and calculated vibrational frequencies ranging from 2000 to 1400 cm^−1^ are presented in Table 3. The B3LYP functional tends to overestimate the fundamental normal modes of vibration, and thus the calculated frequencies were scaled with appropriate values to harmonize the theoretical and experimental wavenumbers [46]. In this study, the scale factor is about 0.97.

The DFT calculation helps assigning vibrational modes to the observed frequencies. The three important vibrations correspond to the two ligands coordinated to the central Ru. There is a clear and strong vibration peak at ∼1840 cm^−1^ that is a stretching vibration for NO in the {Ru(II)-NO^+^} group. The vibration peaks at ∼1560 and ∼1500 cm^−1^ correspond to the vibration of coordinated QN ligands. Monitoring the intensity variation of these peaks offers an important information to investigate the mechanism of the photoinduced reaction of ligand dissociation.

Table 3 lists a comparison of the NO stretching frequencies of the three complexes. Different substituted groups in the 2nd position of the ligand in the [RuCl(2mqn)NO] and [RuCl(hqn)NO] complexes cause a 5 cm^−1^ red shift. This substitution in the [RuCl(2cqn)NO] and [RuCl(hqn)NO] complexes results in a 17 cm^−1^ red shift in the IR absorption peak. Such a shift is clearly a ligand effect; the stretching frequency (*ν*_NO_) of three complexes follows this order: *ν*_NO_ (2cqn) > *ν*_NO_ (2mqn) > *ν*_NO_ (hqn). It is clear that ligand substituents could tune the NO stretching frequency in the three nitrosylruthenium complexes.

### 3.5. Real-Time Measurement of NO Release

**Figure 5:**
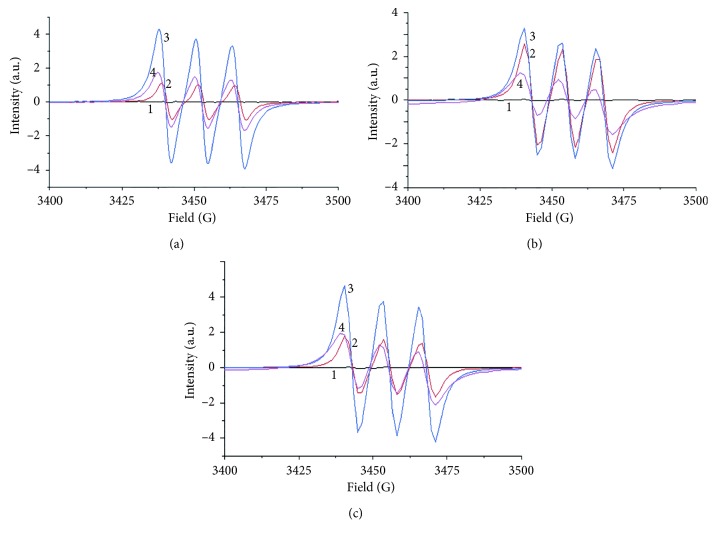
EPR spectra of [RuCl_3_(hqn)NO]^−^ (a), [RuCl_3_(2mqn)NO]^−^ (b), and [RuCl_3_(2cqn)NO]^−^ (c) complexes (1: control, without photoirradiation; 2: 15 s; 3: 30 s; 4: 5 min).

The photoinduced NO release from the three complexes was confirmed with spin-trapping EPR spectroscopy via Fe(MGD)_2_ for detecting NO· in real-time [47, 48].  Figure 5 shows the characteristic triplet signal with a hyperfine splitting constant (hfsc) value of 12.78 G and a *g*-factor of 2.039. These are consistent with published values for NO-Fe^2+^-MGD adducts [49, 50]. It is obvious that free radicals were generated from the complexes with 365 nm photoirradiation, while almost no signal was observed in the dark. The intensity of resulting free radicals increased quickly upon photoirradiation, reaching a maximum at 30 seconds (Figure 5). It then decreased slowly over 5 minutes. Therefore, the NO release could be controlled with photoirradiation, providing the basis for further applications in photobiology and medicine.

### 3.6. NBO Analysis

The natural atomic charges of the three complexes were obtained via natural population analysis (NPA) using the B3LYP method (Table 4). In the {Ru-NO} groups, all N atoms have a net positive charge from 0.451 to 0.467. The electronegative oxygen atoms have negative charges from −0.189 to −0.210, respectively. The calculated Wiberg bond index of NO increases from 1.8449 to 1.8501 and 1.8757 in the order of hqn, 2mqn, and 2cqn complexes. The NO stretching frequency (*ν*_NO_) shifts from 1839.4 to 1844.01 and 1856.5 cm^−1^, which is in agreement with the bond order analyses. The Wiberg bond index of Ru-N decreases from 1.6503 to 1.6408 and 1.6251 for the hqn, 2mqn, and 2cqn complexes, respectively, suggesting that NO is relatively easily released from the 2cqn complex. The results agree with the IR spectral measurements below.

### 3.7. Photoinduced NO Release

**Figure 6:**
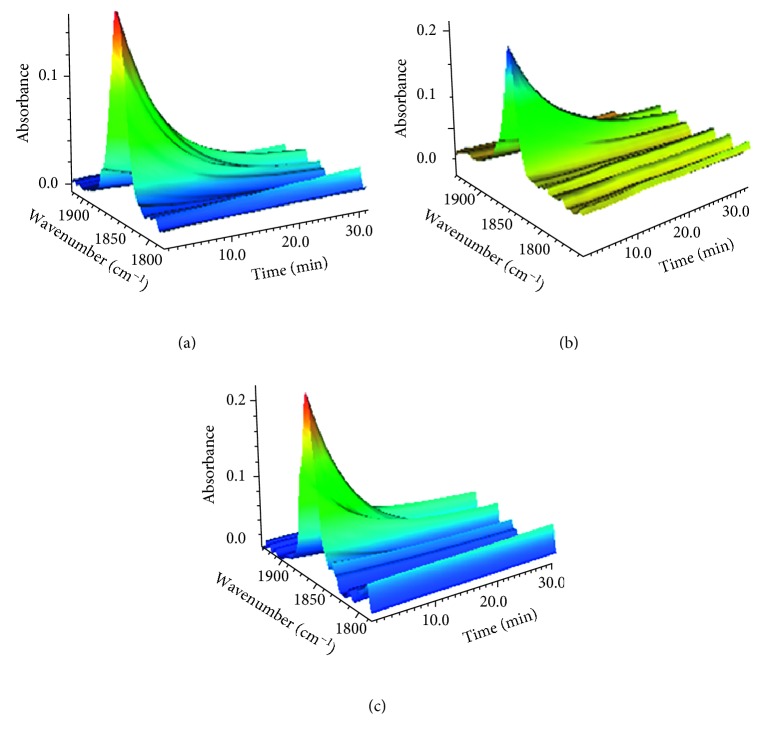
Time resolution of FT-IR spectra of [RuCl_3_(hqn)NO]^−^ (a), [RuCl_3_(2mqn)NO]^−^ (b), and [RuCl_3_(2cqn)NO]^−^ (c) complexes upon photoirradiation.

Next, photoinduced NO release from the three [RuCl_3_(QN)(NO)]^−^ complexes was investigated using time-resolved IR spectra. A series of FT-IR spectra of the NO stretching mode were recorded as a function of photoirradiation.  Figure 6 shows the change in the spectra over time. There is a significant decrease in the intensity of the NO vibrational peak around 1850 cm^−1^, which dominated the photoinduced NO dissociation. The electronic transition from the metal and QN/Cl ligands to the antibonding orbitals of the {Ru(II)-NO^+^} group upon photoirradiation weakens the bonding of Ru-NO and leads to dissociation of NO [51–53]. In addition, the decrease in the NO vibrational intensity for [RuCl(2cqn)NO]^−^ complex is fast relative to the other two complexes, and its half-life of NO dissociation is shorter. Therefore, NO release could be adjusted by complexes using different ligands upon photoirradiation. This strategy can be applied for NO-donor design with potential applications in photobiology and clinical therapy.

Recently, we studied the cytotoxicity and photo-enhanced cytotoxicity of the three [Ru(II)Cl_3_(QN)(NO)]^−^ complexes against HepG-2 cells [27]. The NO free radicals and [Ru(III)Cl_3_(QN)]^−^ complexes resulting from photoirradiation of these complexes are bioactive and cytotoxic and can serve as the potential drugs with dual functions.

## 4. Conclusions

We have shown good agreement between the optimized structural parameters and their crystal structures via DFT calculation at the B3LYP level. The results provide valuable geometrical information and help to assign UV-visible spectra and FT-IR spectra. Meanwhile, DFT calculations for electronic structures and spectral characteristics of [RuCl_3_(QN)(NO)]^−^ complexes provide a better understanding of the photophysical and photochemical properties of these complexes. Real-time NO release was studied via spin trapping of the EPR spectroscopy, and the time-resolved IR spectra showed that three complexes have slightly different half-lives of NO dissociation upon photoirradiation. Moreover, an energy level and component analysis of frontier orbitals shows that the different substituent groups at the 2nd position of the ligands affect their reactivities. This study provides the basis for the design of NO donors for their potential applications in photodynamic therapy.

## Figures and Tables

**Table 1:** Optimized νs. experimental geometries (in Å and °) with 6-311++G(d,p) and Aug-cc-pVDZ-PP as basis set.

	[RuCl_3_(hqn)NO]^−^		[RuCl_3_(2mqn)NO]^−^		[RuCl_3_(2cqn)NO]^−^		X-ray data
	Singlet state	Triplet state	Singlet state	Triplet state	Singlet state	Triplet state	
Ru-N1	2.099	2.087	2.139	2.227	2.154	2.543	2.088
Ru-N2	1.733	1.881	1.734	1.760	1.735	1.758	1.719
Ru-O1	2.011	2.092	1.999	1.981	2.003	1.991	1.993
N2-O2	1.149	1.168	1.148	1.144	1.145	1.144	1.149
∠Ru-N2-O2	177.3	143.2	176.1	177.1	176.0	177.4	174.1

**Table 2:** Relative energies (kcal/mol) and orbital energies (eV) of HOMOs and LUMOs for [RuCl_3_(hqn)NO]^−^, [RuCl_3_(2mqn)NO]^−^, and [RuCl_3_(2cqn)NO]^−^ complexes.

	[RuCl_3_(hqn)NO]^−^	[RuCl_3_(2mqn)NO]^−^	[RuCl_3_(2cqn) NO]^−^
Relative energies (*S* = 0)	−2082.3318613	−2121.6585791	−2541.9469066
Relative energies (*S* = 1)	−2082.291264	−2121.6052187	−2541.8973426
Δ_(S1-S0)_	25.475	33.484	31.102

LUMO	−2.728	−2.708	−2.762
LUMO-HOMO gap	3.207	3.164	3.222
HOMO	−5.935	−5.872	−5.984

**Table 3:** Observed and calculated vibrational frequencies (cm^−1^) and intensities over 2000–1400 cm^−1^ region for [RuCl_3_(hqn)NO]^−^, [RuCl_3_(2mqn)NO]^−^, and [RuCl_3_(2cqn)NO]^−^ complexes.

[RuCl_3_(hqn)NO]^−^		[RuCl_3_(2mqn)NO]^−^		[RuCl_3_(2cqn) NO]^−^		Assignment
Exp.	Cal.	Exp.	Cal.	Exp.	Cal.	
1839.40	1889.44	1844.01	1894.38	1856.55	1906.72	*v*_N=O_ : vs
1576.49	1609.36					*δ*_hqn_ : m
1500.01	1530.48					*δ*_hqn_ : m
1470.07	1491.36					*δ*_hqn_ : m
		1567.78	1593.19			*δ*_2mqn_ : m
		1540.36	1537.52			*δ*_2mqn_ : m
		1507.06	1502.23			*δ*_2mqn_ : m
		1468.27	1472.73			*δ*_2mqn_ : m
				1558.68	1590.61	*δ*_2cqn_ : m
				1493.57	1523.66	*δ*_2cqn_ : m
				1450.55	1470.57	*δ*_2cqn_ : m

**Table 4:** Natural atomic charges and Wiberg bond index of NO in the three [RuCl(QN)NO]^−^ complexes.

Molecule	Peak position/cm^−1^(exp)	Atomic charge		Wiberg bond index	
		N	O	Ru-N	N-O
[RuCl(hqn)NO]^−^	1839.40	0.454	−0.210	1.6503	1.8449
[RuCl(2mqn)NO]^−^	1844.01	0.451	−0.208	1.6408	1.8501
[RuCl(2cqn)NO]^−^	1856.55	0.467	−0.189	1.6251	1.8757
